# Stabilizing the Unstable: Cone Hemiarthroplasty in Geriatric Intertrochanteric Fractures

**DOI:** 10.1016/j.artd.2025.101935

**Published:** 2025-12-30

**Authors:** Arcot Reddy Vamsi Krishna, Babaji Sitaram thorat, Avtar Singh Kamboj, Abhijit das, Kshitij Srivastav, Arshid H. Wani

**Affiliations:** Department of Orthopaedics, Amandeep Hospitals, Amritsar, Punjab, India

**Keywords:** Unstable intertrochanteric fracture, Hemiarthroplasty, Cone stem, Elderly, Bipolar prosthesis, Greater trochanter fixation

## Abstract

**Background:**

Unstable intertrochanteric fractures in the elderly pose a significant treatment challenge due to poor bone quality, comminution, and associated comorbidities. Bipolar hemiarthroplasty offers the advantage of early mobilization and reduced fixation-related complications. This study evaluates short-term outcomes of bipolar hemiarthroplasty using an uncemented titanium fluted, tapered cone femoral prosthesis in such fractures.

**Methods:**

A retrospective analysis was conducted on 43 consecutive elderly patients treated with uncemented bipolar hemiarthroplasty using a tapered, fluted cone stem between June 2023 and July 2024. Radiographic parameters—including stem subsidence, greater trochanter union, and limb-length discrepancy—were assessed at serial follow-ups. Functional outcomes were evaluated using the Harris Hip Score.

**Results:**

Of the 43 patients operated on, 40 completed a minimum of 12 months follow-up. Greater trochanteric union was observed in 97.7% of cases, with one persistent nonunion causing abductor weakness and early dislocation. Mean stem subsidence was 3.5 mm (0.5–20 mm), with all settling occurring within the first 3 postoperative months; 4 patients (9.3%) experienced subsidence of 5 mm or more, including one requiring revision. The mean limb-length discrepancy was 4.7 mm (1–10 mm). At final follow-up, the mean Harris Hip Score among surviving patients was 91.28, with 29 patients (72.5%) achieving excellent outcomes and 11 patients (27.5%) achieving good outcomes.

**Conclusions:**

Bipolar hemiarthroplasty using a tapered, fluted cone stem appears to be a reliable option for carefully selected elderly patients with unstable intertrochanteric fractures, offering predictable fixation, early weight-bearing, and favorable short-term functional results.

## Introduction

Intertrochanteric fractures represent one of the most frequent and life-threatening injuries among the elderly [1]. As the population ages, the prevalence of osteoporosis is rising, further complicating the management of unstable intertrochanteric fractures. These injuries often involve significant displacement or comminution, particularly in elderly individuals with poor bone quality. They are frequently associated with complications such as nonunion, implant failure, and femoral head perforation [2,3].

The primary treatment goal is to restore the patient’s preinjury mobility and independence at the earliest. Currently, there is an ongoing debate regarding the optimal management approach—whether to opt for internal fixation or proceed with hip arthroplasty in elderly osteoporotic patients [4,5]. Bipolar hemiarthroplasty has gained recognition as a viable option for these fractures, as it facilitates early full weight-bearing and functional recovery [6]. Additionally, by allowing earlier mobilization compared to internal fixation, hemiarthroplasty may reduce the incidence of postoperative complications such as deep vein thrombosis, pulmonary embolism, and cerebrovascular events [7].

A critical question in arthroplasty for these fractures is the choice between cemented and cementless femoral stems. While cemented stems provide strong initial fixation, intraoperative cardiovascular instability associated with cement use is now understood to result from multifactorial physiological responses rather than the cement material itself [8]. Cementless stems, in contrast, avoid the need for cement pressurization and achieve durable fixation through biological osseointegration. These implants also offer the advantages of shorter operative time and reduced cardiopulmonary stress, making them particularly suitable for elderly patients with multiple comorbidities [9]. Advances in stem design have further improved their biomechanical stability and adaptability, although consensus on the optimal stem type for unstable intertrochanteric fractures in osteoporotic bone remains elusive.

This study aims to assess the short-term outcomes of using a titanium fluted, tapered, cone-shaped cementless femoral stem in bipolar hemiarthroplasty for treating unstable intertrochanteric fractures in the geriatric population.

## Materials and Methods

### Patient selection

**Figure 1:**
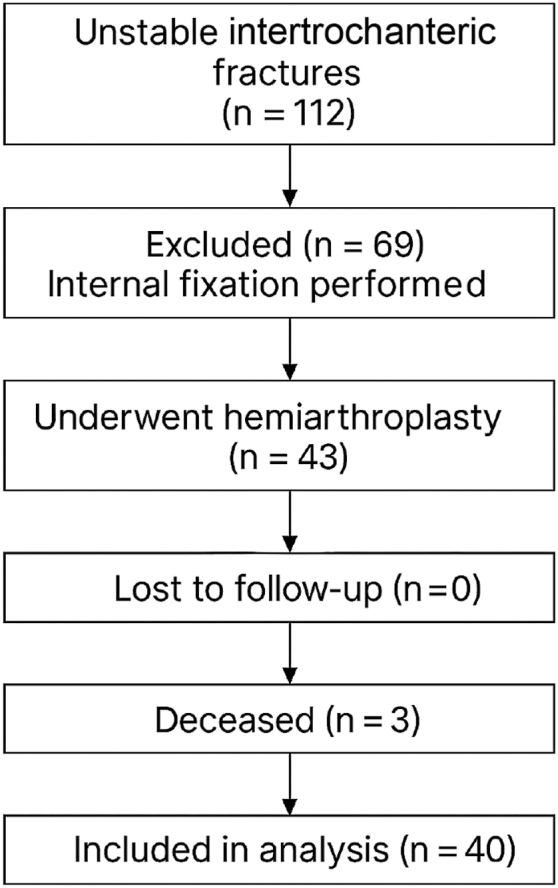
Flow diagram of the patient selection and follow-up.

Between June 2023 and July 2024, all consecutive patients diagnosed with unstable intertrochanteric fractures who met the predefined inclusion criteria underwent primary bipolar hemiarthroplasty using a cone femoral prosthesis at Amandeep Hospitals, Amritsar (Table 1). During the same period, a total of 112 patients with unstable intertrochanteric fractures were treated at our institution, of whom 43 (38.4%) underwent hemiarthroplasty, while 69 (61.6%) were managed with internal fixation. A flow diagram summarizing patient selection, treatment allocation, follow-up status, and the final number of patients included in the analysis is presented in Figure 1.

**Table 1:** Demographic characteristics of the study population.

Demographic characteristic	Data
No of patients (n)	43
Mean age (y)	83.6 (range, 70-106)
Gender	
Males	11 (25%)
Females	32 (75%)
Type of fracture (according to AO classification)	
31- A2.2	32
31- A2.3	9
31- A3.3	1
31- A1.2	1
Side of injury	
Right side	22
Left side	21
Comorbidities	
None	12
Hypertension and diabetes	13
Multiple comorbidities	18
Mean time from admission to surgery	2.3 d

Treatment allocation followed a uniform departmental protocol for managing unstable fracture patterns in elderly patients. The cohort was followed for a mean duration of 18 months (range, 13–26 months). Patients aged 70 years or older with radiologically confirmed unstable intertrochanteric fractures classified as Arbeitsgemeinschaft für Osteosynthesefragen / Orthopaedic Trauma Association 31-A2.2, A2.3, or A3 were included. Those with pathological fractures or polytrauma were excluded. Clinical evaluation was performed using the Harris Hip Score (HHS), and serial radiographs were obtained at each follow-up visit to assess fracture healing and stem stability.

### Implant used

The femoral stem employed was a Wagner cone-type fluted stem (Vulcan Stem, Pitkar Pvt. Ltd.), made of uncemented monoblock titanium alloy. It features a grit-blasted surface to facilitate long-term biological fixation via osseointegration. The implant is designed with a 5-degree taper and 8 longitudinal ribs, providing rotational stability and press-fit distal fixation. It is available in 2 neck angles (125° and 135°), allowing surgeons to restore important biomechanical parameters, including the center of rotation, offset, and leg length.

### Surgical technique

All patients underwent a preoperative evaluation, including assessment of the injury mechanism, comorbid conditions, and cardiovascular status, in consultation with a physician. Following appropriate anesthetic clearance, surgeries were performed under spinal or general anesthesia.

The patient was positioned in the lateral decubitus position with the affected limb facing upward. After standard skin preparation and draping, a posterolateral incision was made. Subcutaneous and deep tissue dissection was performed to expose the fracture site.

• If the greater trochanter (GT) was involved in the fracture, a transtrochanteric or interfragmentary approach was employed. In such cases, the limb position was not altered during the procedure, and femoral anteversion was determined intraoperatively with reference to the posterior condylar axis in the lateral position.
• If the GT was intact, a modified Southern approach was used, with short external rotators detached from the intertrochanteric line using electrocautery, and the joint capsule incised along the femoral neck. In this approach, the hip was flexed, adducted, and internally rotated, with the knee flexed to 90 degrees. Anteversion was then assessed, referencing the adjusted limb position.

**Figure 2:**
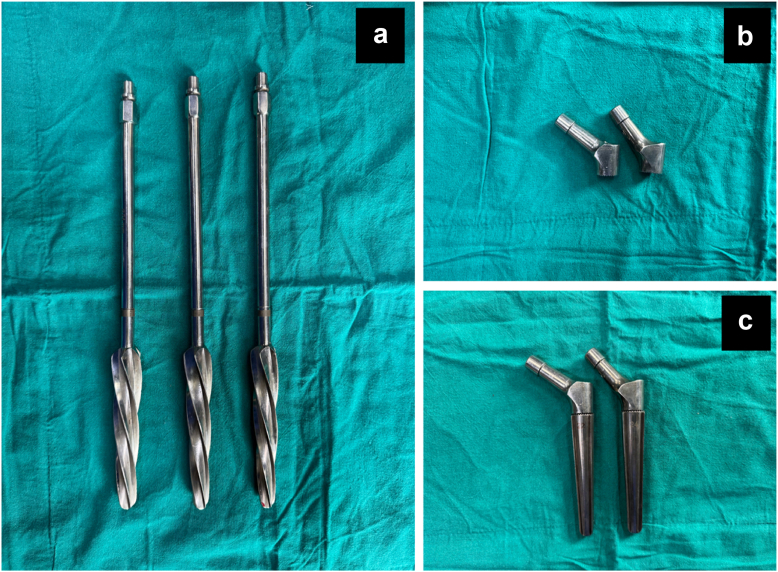
Tapered reamers and trial cone stems with different neck–shaft angle options used during hemiarthroplasty. (a) Different diameter reamers, (b) and (c) Trial implants with 125 and 135 degree neck–shaft angle.

Following femoral head and neck excision, the head size was estimated using a trial template. The medullary canal was progressively broached with tapered reamers along the femur's length. Reaming continued until noticeable resistance was felt, ensuring a proper fit for press-fit fixation. A trial stem was inserted to assess alignment and fit. Representative intraoperative photographs demonstrating the tapered reamers and trial cone stems with different neck–shaft angles are shown in Figure 2.

**Figure 3:**
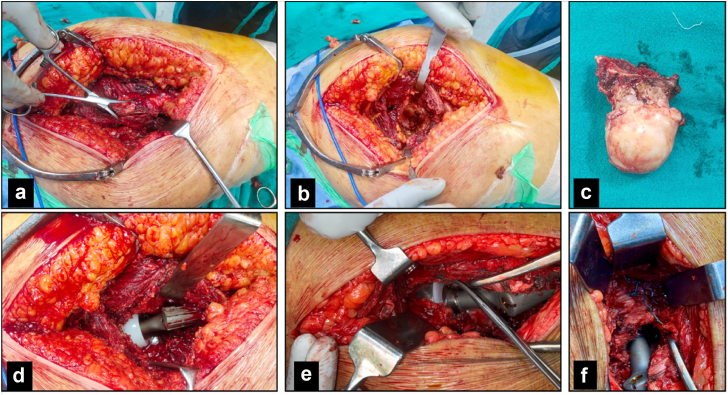
Stepwise operative technique illustrating cone hemiarthroplasty for unstable intertrochanteric fractures (a) Transtrochanteric or interfragmentary exposure of the fracture. (b) Exposure of the proximal femur and fracture configuration. (c) Resected femoral head and neck with associated calcar fragment. (d) Trial reduction after insertion of the cone stem prosthesis. (e) Identification of the hip capsule for anatomic repair. (f) Completed capsular repair before closure.

The cone prosthesis was then placed using its dedicated instrumentation, aiming for approximately 10–20° of femoral anteversion. The prosthesis was rotated into the desired version and impacted into its final position with moderate mallet blows. Final seating depth was checked against preoperative templating, followed by insertion of the appropriately sized bipolar cup and head component. The hip was reduced, and intraoperative checks for stability, range of motion, and leg length discrepancy were performed (Fig. 3).

**Figure 4:**
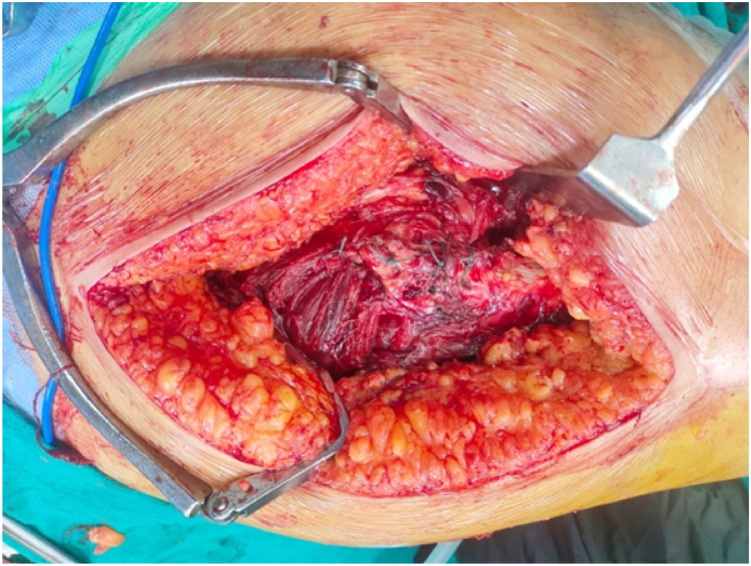
Suture-based fixation of the greater trochanter (Ethibond).

Greater trochanteric fragments were primarily fixed with nonabsorbable sutures (Ethibond) (Fig. 4).

**Figure 5:**
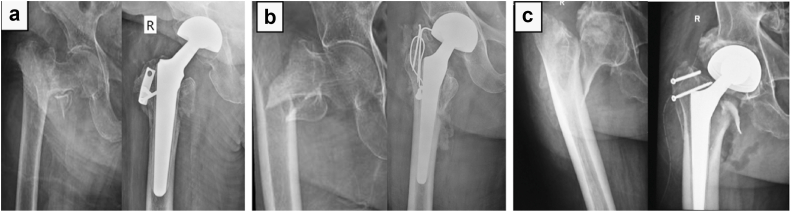
Use of different methods for fixation of the greater trochanter. (a) Fixation using a plate, (b) Fixation using TBW, and (c) Fixation using screws. TBW, tension band wiring.

In selected cases, tension band wiring, plate augmentation, or screws were employed as needed based on fracture morphology (Fig. 5).

**Figure 6:**
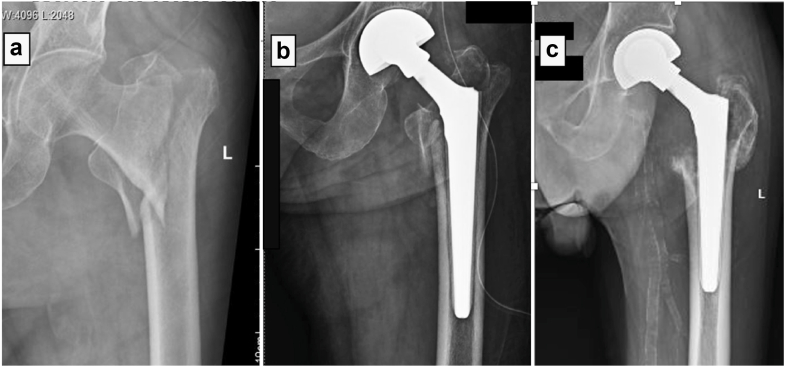
Radiographs of a patient who underwent bipolar hemiarthroplasty using a titanium, fluted, tapered cone prosthesis. (a) Preoperative radiograph of the involved side, AP view. (b) Immediate postoperative radiograph. (c) Radiograph at the latest follow-up. AP, anteroposterior.

All patients were mobilized from postoperative day one with tolerable weight-bearing using a walker. Drain removal and the first dressing change were performed on postoperative day 2. Thromboembolic prophylaxis consisted of low-molecular-weight heparin during the hospital stay, along with intermittent pneumatic compression devices while in bed. Patients were discharged once medically optimized and cleared by physiotherapy and were sent home with oral antiplatelet therapy (Ecosprin) for 6 weeks. Follow-up was arranged on the fourteenth postoperative day for suture removal and subsequently at monthly intervals for clinical and radiological evaluation (Fig. 6). Mortality status and functional outcomes were obtained from hospital electronic records, outpatient visits, and telephonic contact with patients or their family members. Patients who died during the follow-up period were excluded from functional outcome analysis, and HHSs were recorded for all surviving patients who attended their final follow-up visit.

## Results

A total of 43 patients were included in the study, comprising 11 males (25%) and 32 females (75%), with a mean age of 83.6 years (range, 70–106). The mean operative time was 71.7 minutes (range, 50–95 minutes), and the mean intraoperative blood loss was 187.4 ml (range, 150–250 ml). Twenty-two procedures were performed on the right side and 21 on the left. Fractures were classified as Arbeitsgemeinschaft für Osteosynthesefragen / Orthopaedic Trauma Association 31-A2.2 in 32 patients, 31-A2.3 in 9, 31-A3.3 in 1, and 31-A1.2 in 1 patient. The mean time from admission to surgery was 2.3 days, and the mean hospital stay was 6.33 days (range, 3–14 days).

GT fixation was performed using Ethibond sutures in 39 patients (90.7%), tension band wiring in 2 (4.7%), screw fixation in 1 (2.3%), and plate fixation in 1 (2.3%). Radiographic evaluation demonstrated successful GT union in 42 of 43 patients (97.7%). One patient demonstrated persistent GT nonunion with associated abductor insufficiency, which contributed to an early postoperative dislocation.

Radiographically, the mean femoral stem subsidence was 3.5 mm (range, 0.5–20 mm). All subsidence occurred within the first 3 postoperative months, with no progression at the 6-month or 1-year follow-up. Subsidence ≥5 mm was observed in 4 patients (9.3%), including 2 patients (4.7%) with marked early subsidence >10 mm. One of these patients experienced recurrent dislocation and underwent open reduction with femoral component revision. The second patient, despite demonstrating 12 mm of early subsidence, remained clinically asymptomatic and exhibited stable radiographs and satisfactory function throughout 1-year follow-up.

The mean postoperative limb-length discrepancy was 4.7 mm (range, 1–10 mm), and no patient demonstrated a clinically significant discrepancy greater than 10 mm.

Two patients (4.7%) experienced postoperative dislocation. One dislocation was associated with marked early stem subsidence (>10 mm), whereas the other occurred in the patient with GT nonunion and abductor insufficiency. Both patients were managed surgically with no recurrence. One patient (2.3%) developed a superficial surgical site infection, which resolved with wound wash, debridement, and intravenous antibiotics. No cases of deep infection or periprosthetic joint infection were encountered.

Three patients (7.0%) died during the follow-up period. Mortality was confirmed through hospital records and telephonic verification with family members. These patients were unable to attend the 1-year follow-up visit. All 40 surviving patients were able to complete the HHS assessment at final follow-up; none had cognitive impairment severe enough to preclude functional evaluation, and caregiver assistance was used when necessary.

At final follow-up, the mean HHS was 91.28 ± 5.07. Among the 40 surviving patients, 29 (72.5%) achieved excellent outcomes (HHS ≥90), and 11 (27.5%) achieved good outcomes (HHS 80–89). No patient had a fair or poor functional result.

## Discussion

Unstable intertrochanteric fractures in the elderly remain a significant therapeutic challenge due to the absence of medial cortical support, significant fracture displacement, reverse obliquity patterns, extensive comminution, and underlying osteoporosis, all of which reduce the reliability of internal fixation [[10], [11]]. Numerous studies have reported high failure rates with cephalomedullary nails in osteoporotic, unstable patterns—particularly A2.2, A2.3, and reverse obliquity fractures—due to varus collapse, cut-out, and delayed mobilization [[12], [13], [14]]. Such complications are especially detrimental in frail older adults who are unable to tolerate prolonged partial weight-bearing or revision procedures. Bipolar hemiarthroplasty offers the advantage of immediate structural stability, allowing early weight-bearing and improved functional recovery [9].

The choice of femoral stem plays a pivotal role in determining the stability and longevity of a hemiarthroplasty construct, particularly in elderly patients with poor bone stock. In our series, the use of a tapered, fluted, uncemented cone stem provided predictable fixation and allowed an early postoperative rehabilitation protocol, reflected by the predominantly excellent or good functional outcomes at 1 year. The tapered cone design, inspired by the Wagner philosophy, provides axial stability through its 5° conical geometry and rotational stability through its longitudinal flutes [15]. Prior studies have demonstrated that such stems achieve reliable metaphyseal fixation even in osteoporotic femora, with early osseointegration facilitated by a grit-blasted surface. The circular cross-section of the stem also allows the surgeon to freely adjust femoral anteversion, a notable advantage in unstable fractures where native anatomy is distorted or when trochanteric approaches alter the proximal femoral entry point [16]. Consistent with published literature, our series did not encounter any intraoperative fractures or aseptic loosening, supporting the suitability of this stem design in fracture arthroplasty.

Subsidence patterns in our cohort followed the well-described behavior of tapered, cementless stems, with all migration occurring within the first 3 months and ceasing thereafter. This early subsidence represents settling of the implant until sufficient axial and rotational stability is achieved through bony on-growth. Prior arthroplasty studies report early subsidence ranges between 1 and 4 mm in conical stems, with occasional outliers exceeding 5 mm, particularly in osteoporotic bone or undersized stems [17]. Our mean subsidence (3.5 mm) aligned well with these findings. Notably, 2 patients experienced marked early subsidence >10 mm. One of these developed recurrent dislocation and required revision of the femoral component. The second, despite showing 12 mm of early subsidence, remained clinically asymptomatic and radiographically stable throughout follow-up—an observation also described in other series, where substantial radiographic subsidence does not always correlate with functional disability when the stem settles into a stable position [18].

GT integrity is critical for restoring abductor function and maintaining hip stability. Existing reports emphasize that trochanteric nonunion or insufficient fixation is associated with postoperative limp, reduced HHSs, and an increased risk of dislocation [19]. In our study, the majority of patients (90.7%) underwent trochanteric fixation using nonabsorbable Ethibond sutures, which provided adequate stability for biological healing. The simplicity and minimal invasiveness of this method, along with its proven utility in soft tissue reattachment, made it the preferred option. In select cases with severe comminution or displacement, adjunct fixation techniques such as tension band wiring, screws, or plate augmentation were used [20]. In our cohort, greater trochanteric union was obtained in 97.7% of cases, and these patients maintained good abductor function without clinically meaningful lurch. The single case of GT nonunion (2.3%) resulted in abductor insufficiency. It was a key factor in the patient’s early dislocation, consistent with reports identifying failed GT healing as a major driver of postoperative hip instability. The overall dislocation rate in our study (4.7%) is comparable to previously reported rates ranging from 2% to 10% following hemiarthroplasty for intertrochanteric fractures [21].

Early mobilization is a cornerstone of geriatric fracture management, reducing the risk of pneumonia, thromboembolic events, deconditioning, and dependency. With a stable implant construct, our patients were able to begin assisted weight-bearing from postoperative day one and achieved full weight-bearing at a mean of 3.4 weeks, which likely contributed to the high mean HHS (91.28 ± 5.07) observed at final follow-up.

This study has several limitations that deserve consideration. First, it is a single-center, retrospective series with a relatively small cohort, and the patients included were a protocol-selected group who were considered less suitable for internal fixation because of fracture pattern, bone quality, or preinjury functional status. Therefore, the findings are most applicable to this specific clinical subgroup and should not be broadly generalized to all elderly patients with intertrochanteric fractures. Although earlier literature supports the use of hemiarthroplasty in selected unstable fracture patterns, very few reports have specifically evaluated the performance of a tapered, fluted cone stem in this context. While our study was not designed as a direct comparison with internal fixation, it highlights the practical feasibility and encouraging short-term outcomes of this implant in complex fractures. These findings provide a useful benchmark and can help guide future matched or randomized studies exploring how this approach may complement or outperform internal fixation in carefully chosen patients. Finally, our follow-up period is relatively short-, and longer-term outcomes, including implant survival, late loosening, acetabular wear, and periprosthetic fractures, warrant further investigation.

## Conclusions

Bipolar hemiarthroplasty using an uncemented tapered cone femoral prosthesis is a reliable and effective option for managing unstable intertrochanteric fractures in the elderly. The implant offers stable fixation even in osteoporotic bone, facilitates early mobilization, and demonstrates favorable short-term functional outcomes with minimal complications. Proper fixation of the GT and adherence to postoperative rehabilitation protocols are essential to optimize hip stability and abductor function. Given its safety profile and ability to restore mobility in a frail population, this technique represents a valuable alternative to internal fixation in selected patients.

## Ethics approval and consent to participate

The current study was approved by the Institute Ethics Committee, Amandeep Hospitals, Amritsar (Ref. No: AMAN/EC/08/14). Written informed consent was obtained from all participants included in the study.

## CRediT authorship contribution statement

**Arcot Reddy Vamsi Krishna:** Writing – original draft, Methodology, Formal analysis, Data curation, Conceptualization. **Babaji Sitaram thorat:** Writing – review & editing, Methodology, Formal analysis, Conceptualization. **Avtar Singh Kamboj:** Writing – review & editing, Supervision, Methodology, Investigation, Formal analysis, Conceptualization. **Abhijit das:** Writing – review & editing, Methodology, Investigation, Data curation. **Kshitij Srivastav:** Writing – review & editing, Supervision, Investigation, Conceptualization. **Arshid H. Wani:** Writing – review & editing, Supervision, Methodology, Investigation, Data curation.

## Conflicts of interest

The authors declare there are no conflicts of interest.

For full disclosure statements refer to https://doi.org/10.1016/j.artd.2025.101935.

## Appendix A Supplementary data

Conflict of Interest Statement for Das

Conflict of Interest Statement for Wani

Conflict of Interest Statement for Kamboj

Conflict of Interest Statement for Thorat

Conflict of Interest Statement for Srivastav

Conflict of Interest Statement for Krishna
